# Effectiveness of Resilience Interventions on Psychosocial Outcomes for Persons With Neurocognitive Disorders: A Systematic Review and Meta-Analysis

**DOI:** 10.3389/fpsyt.2021.709860

**Published:** 2021-08-19

**Authors:** Ying Wang, Iris Chi, Yuning Zhan, Wenjang Chen, Tongtong Li

**Affiliations:** ^1^School of Philosophy and Sociology, Lanzhou University, Lanzhou, China; ^2^Evidence Based Social Science Research Center, Lanzhou University, Lanzhou, China; ^3^Suzanne Dworak-Peck School of Social Work, University of Southern California, Los Angeles, CA, United States; ^4^Edward R. Roybal Institute on Aging, University of Southern California, Los Angeles, CA, United States; ^5^School of Economics and Management, Hangzhou Normal University, Hangzhou, China

**Keywords:** resilience, intervention, meta-analysis, psychosocial outcomes, neurocognitive disorders

## Abstract

**Background:** Neurocognitive disorders, such as mild cognitive impairment (MCI), dementia, and Alzheimer's disease, not only harm people's cognitive function but also lead to negative emotions, poor quality of life (QOL), and unsatisfactory level of well-being. Resilience can be defined as a dynamic and amendable process, which maintains or improves life satisfaction and quick recovery from own dilemma. However, no meta-analysis of randomized controlled trials (RCTs) has thus far examined the effectiveness of resilience interventions among persons with neurocognitive disorders, and the results of RCTs were inconsistent. This systematic review aimed to assess the effectiveness of resilience interventions on psychosocial outcomes among persons with neurocognitive disorders.

**Methods:** Nine electronic Chinese and English databases (the Cochrane Library, PsycINFO, Web of Science, PubMed, Medline, Eric, JSTOR, CNKI, and WANGFANG) were searched through April 2021. Only RCTs were included, and the quality of the included studies was assessed by the Cochrane “Risk of Bias” tool. Meta-analysis was carried out on psychosocial outcomes, and heterogeneity was investigated by subgroup and sensitivity analysis. RevMan 5.4 was used for meta-analysis.

**Results:** Fourteen RCT studies were identified, representing a total of 2,442 participants with neurocognitive disorders. The risk of bias was high or unclear for most included studies in the domains of allocation concealment, blinding participants, and interventionists. Meta-analysis showed that heterogeneity was low or moderate. There were significant differences in favor of resilience interventions compared with control on the outcome of QOL, using the Quality of Life-Alzheimer Disease scale (QOL-AD) [*I*^2^ = 36%, standardized mean difference (SMD) = 0.14, 95% CI (0.02, 0.26), *p* = 0.02], and no significant differences on depression, using the Cornell Scale for Depression in Dementia (CSDD) [*I*^2^ = 41%, SMD = −0.14, 95% CI (−0.34, 0.05), *p* = 0.16], and neuropsychiatric symptoms using the Neuropsychiatric Inventory Questionnaire (NPI-Q) [*I*^2^ = 62%, SMD = −0.10, 95% CI (−0.37, −0.16), *p* ≤ 0.46].

**Conclusions:** Resilience interventions had a significant benefit on QOL but no significant benefit on depression and neuropsychiatric behavioral symptoms. More evidence is needed to answer questions about how to implement resilience interventions and how to evaluate their effectiveness.

## Introduction

Mild cognitive impairment (MCI), dementia, or Alzheimer's disease (following abbreviation neurocognitive disorders) are chronic progressive syndrome. During the phase transition process, divergent sections of the brain are affected, and a persons' capability of adaptation to the disease and environment gradually decreases (1). Neurocognitive disorders not only impair the person's memory, orientation, thinking, cognitive functioning, and language (2), but also trigger emotions and psychological symptoms, such as depression and anxiety (3, 4). In addition, the impact of neurocognitive disorders is long-lasting; it is extremely exacting or nearly impossible to be cured completely (5, 6). With these detrimental outcomes, neurocognitive disorders further lead to an unsatisfactory level of well-being and quality of life (QOL), such as physical function, and financial instability of individuals and families; these results also undermine people's ability to fulfill family, social, and professional roles (7). Neurocognitive disorders currently affected tens of millions of people all over the world and caused enormous medical and economic burdens. For example, there were 50 million people with dementia worldwide (2), and the number of people with dementia worldwide is projected to be 152 million in 2050 (8). Dementia contributes significantly to the global burden of disease, costing an estimated $818 billion annually (9), expected to reach $2 trillion by 2030 (8, 10–12). Thus, neurocognitive disorders are regarded as one of the greatest social, health, and economic challenges of the twenty-first century (8, 11–13). It is progressively crucial to develop strategies that facilitate and help persons with neurocognitive disorders to maintain independence, well-functioning, and high QOL in the long run.

There were various approaches to coping with the challenges affiliated with neurocognitive disorders. Resilience-centered interventions can be seen as one important approach to adapting to stress and reducing the adverse impact of the stressors (7). Luthar and Cicchetti (14) defined resilience as “a dynamic and amendable process,” in which people use resources to acclimate to adversity (15). Meanwhile, Kunzler et al. (16) highlighted that resilience-centered interventions could be seen as the process of maintenance, withstanding, overcoming, adjustment, adaptation, posttraumatic growth, stress-related growth, rebound from a stressor, or rapid readjustment. As mentioned above, resilience can be defined as a process: after experiencing acute (short-run) or chronic (long-run) issues on health and stress, an individual can actively adapt, withstand, overcome, adjust, cope, and grow to maintain and improve his or her QOL with the support from multifaceted resources on individual and social levels.

The literature illustrated that protective factors of resilience were diverse, such as self-care, adherence to treatment programs, patient perceptions of pain and disease, adherence to physical activities, self-empowerment, health-related QOL, self-efficacy improvement, stress, depression, and anxiety reduction, optimism in viewpoints, and recovery acceleration (17–21). However, the process of resilience interventions focuses on reinforcing personal characteristics and exterior assets in response to a severe challenge to build an inclusive environment with multifaceted psychosocial supports (22). Similarly, other literature also suggested that resilience framework should include individual, family, community, and social components (23, 24). For example, Harris (24) stressed strengthening personal attributes in the process of resilience interventions, which could include self-acceptance of a person with neurocognitive disorders with the shifts in self, nurturing the individual's remaining competence, a positive perspective on diseases and dilemmas, and recognition of numerous means in which someone with dementia can contribute meaningly to their friends, family, and/or the community. Casey et al. (22) suggested five domains to implement resilience interventions: having a “fighting spirit” and personal control, maintaining solid family relationships, maintaining ties to communities, increasing awareness, addressing negative attitudes through dementia education, and engaging in physical activity. Kunzler et al. (16) suggested that resilience should include supportive doctors, linkages to helpful community groups and events, and sympathetic and supportive social surroundings. Overall, the literature mentioned above indicated that in the resilience process, multifaceted interventions should be taken, involving interactions between individuals and the external resources.

The outcomes of resilience-centered interventions are psychosocial, such as improving QOL, restoring normal performance, maintaining mental health, improving adaptability (25), better adjustment (26), enhancing mental well-being (27), reducing care dependence, good social relations, positive self-image (28), reducing burden or stress (27, 29), enhancing intent or meaning of life, and obtaining self-esteem, positive emotions, self-efficacy, boldness, active coping, optimism, social support, adaptation, and cognitive flexibility (including positive reassessment and acceptance) (16). However, one question arises regarding how to measure resilience-centered intervention outcomes in different resilience approaches. Windle et al. (30) studied tools for resilience interventions and concluded that the conceptual and theoretical adequacy of the scales was questionable, with no existing “gold standard” of resilience measures. Whelan et al. (15) indicated that key sets of outcomes for resilience in neurocognitive disorders have not been identified. For example, Ghanei Gheshlagh et al. (7) used three scales, which are Resilience Scale-25 (RS-25), Connor–Davidson Resilience Scale-10 (CDRISC-10), and Connor–Davidson Resilience Scale-25 (CDRISC-25), to assess the effectiveness of the resilience process for people with chronic physical diseases. Saint-Bryant et al. (26) used the Cornell Scale for Depression in Dementia (CSDD), Quality of Life Alzheimer's Disease scale (QOL-AD), and the Index of Relocation Adjustment Scale (IRA) to measure depression, QOL, and adjustment conditions as the outcomes of resilience process for older adults with dementia.

Another question is that the effect of resilience interventions is inconclusive. Thus far, there are only three systematic reviews (31–33) related to resilience interventions or outcome measures. Although Li et al. (31) claimed to contain resilience training, it was a mere strength training different from resilience. They also assessed cognitive outcomes, such as executive cognitive ability, global cognitive function, memory, and attention. Findings indicated positive effects on the executive cognitive capability and overall cognitive function, a weak-positive effect on memory, and no significance in attention. In the review of Carrion et al. (32), the resilience interventions focused on cognition-oriented caregiving approaches. The included 47 randomized controlled trials (RCTs) did not conduct a meta-analysis, and the results were inconclusive. In the review of Regan and Varanelli (33), the resilience interventions used modified cognitive behavior therapy (CBT) and problem-solving approach. They assessed three outcomes, including depression, anxiety, and adjustment in older adults with mild cognitive impairment and early dementia. The included seven RCTs and eight pre–post studies indicated positive effects in reducing depression in older persons with early dementia. However, Regan and Varanelli's (33) review did not conduct a meta-analysis, so it was unable to draw a clear conclusion about the intervention effect because of the divergent methods of the included studies. Additionally, the narrative review (15) identified five resilience interventions in three empirical studies of six papers, including Peer Support Network Services, Dementia Advisors, Memory Makers, Visual Arts Enrichment Activities, and Early-Stage and Beyond Community Activities. However, this narrative review included empirical studies, and the effectiveness of the resilience interventions could not be determined due to the study design.

Overall, there exist several dissimilarities regarding methodology and quality of studies among previous systematic reviews, which leads to inconsistent results regarding the effectiveness of resilience interventions. Currently, there is no systematic review that both includes RCT studies and conducts a meta-analysis. Therefore, this review aimed to identify RCT resilience interventions among persons with neurocognitive disorders to assess the effectiveness and provide further detailed evidence. This may contribute to enhancing existing resilience interventions and to facilitating the future development of such programs.

## Methods

### Criteria for Considering Studies for This Review

#### Participants

Participants were people of all ages with neurocognitive disorders, including dementia, mild cognitive impairment (MCI), and Alzheimer's disease. Participants' formal diagnoses on types and severity of those neurocognitive disorders were based on corresponding scales, including the Diagnostic and Statistical Manual of Mental Disorders, Fifth Edition (DSM-5) (34); International Statistical Classification of Diseases and Related Health Problems, 10th Revision (ICD-10) (35); or other comparable diagnostic criteria. We included people living in diverse settings, such as the community, hospitals, and nursing homes. We did not use a criterion for age so as not to exclude studies in which some participants were below 60 years old. If a mixed sample of participants (e.g., people with dementia and their caregivers) were found and the data of persons with dementia were reported separately or were collected by contacting the author, these studies also were included.

#### Interventions

Any intervention that promotes a person's state of adaptation and adjustment with the help of personal attributes and external assets, regardless of content, duration, setting, or mode of delivery, was included. Resilience, for example, can include active coping (e.g., planning, problem solving), self-efficacy, optimism or positive attributional style, cognitive flexibility (e.g., positive reassessment and acceptance of negative emotions and conditions), religiosity and spirituality (e.g., frequent religious visits), positive emotions or positive affect, hardiness, self-esteem, intent or meaning of life, sense of coherency (internal), locus of control, coping flexibility, hope, humor, altruism (16), physical strength training, formal or informal care, social connection, and community or other external resource support.

Studies were excluded if they involved animal trials and non-psychological or non-social interventions of resilience, such as pharmacological interventions (e.g., treatment with antidepressants).

#### Comparators

Comparators included no treatment, treatment as usual (TAU) (e.g., routine medication and usual social activities), and wait-list control. If the control group adopted active control, such as music, physical, and cognitive–behavioral, rather than no treatment, TAU, or wait-list control, the literature was excluded. For studies with two or more controls, our meta-analysis was conducted only using the control group of no treatment, TAU, or wait-list control.

#### Outcome Measures

We defined outcomes as assessments of psychosocial adaptation. For these outcomes, QOL was a primary outcome, and others were secondary outcomes, such as social relations, positive self-image, self-efficacy, hardiness, anxiety, and depression. We accepted all psychosocial assessment tools used in the included studies. Outcomes were assessed before the treatment, upon completion of the treatment, and follow-up evaluations to assess long-term effects. We considered measures self-assessed and scored by observers or clinicians.

Studies were excluded if the studies contained non-psychosocial outcomes of resilience, such as brain structure, immediate memory, attention and calculation, deferred memory, time orientation, location orientation, language, visual space, or the geographical environment. This ensured that the review focused on the psychosocial outcomes of resilience interventions. The absence of the outcome values was an exclusion criterion for this review: if the values of mean and standard deviation (SD) were not reported in the description of outcome, mean and SD cannot be obtained by contacting the authors, or mean and SD cannot be calculated by the review manager software or calculator provided by Cochran, the original study was deleted (Review manager software or calculator: https://training.cochrane.org/online-learning/core-software-cochrane-reviews/revman/revman-5-download; https://training.cochrane.org/resource/revman-calculator).

#### Types of Studies

Our review intended to include both published and unpublished RCTs in Chinese or English language. We also took into account cluster RCTs.

### Electronic Searches

Nine electronic databases (the Cochrane Library, PsycINFO, Web of Science, PubMed, Medline, Eric, JSTOR, CNKI, and WANGFANG) were searched through April 2021. Gray literature was also searched from ProQuest Dissertations & Theses Database (PQDT) and DUXIU and reviewed. Authors of relevant conference abstracts were reached out for possible information sharing. The search terms used were the following: (a) dementia or Alzheimer or cognitive loss: MCI or dementia or Alzheimer or ADRD or “cognitive impai^*^” or “cognitive loss” or “cognitive decline”; (b) resilience or resiliency: resilien^*^ OR adjust^*^ OR adapt^*^ OR “post-traumatic growth” OR “post-traumatic growth” OR “stress-related growth” OR withstand^*^ OR overcom^*^ OR resist^*^ OR recover^*^ OR thriv^*^ OR adapt^*^ OR adjust^*^ OR bounc^*^ back; and (c) RCT or random^*^. We used “subject OR title OR abstract OR keywords OR topic” to search. Search strategy was (MCI or dementia or Alzheimer or “cognitive impai^*^” or “cognitive loss” or “cognitive decline”) AND (resilien^*^ OR adjust^*^ OR adapt^*^ OR “post-traumatic growth” OR “post-traumatic growth” OR “stress-related growth” OR withstand^*^ OR overcom^*^ OR resist^*^ OR recover^*^ OR thriv^*^ OR adapt^*^ OR adjust^*^ OR bounc^*^ back) AND (RCT or random^*^).

### Assessment of Risk of Bias in Included Studies

We employed the Cochrane “Risk of Bias” tool (36) to identify any risk of bias with a judgment of low risk, high risk, or unclear risk of bias for each study of the following areas: (1) selection bias, (2) random sequence generation, (3) allocation concealment, (4) blinding of participants and personnel, (5) blinding of outcome assessment, (6) incomplete outcome data, and (7) selective reporting.

### Data Collection and Analysis

#### Studies Screening

Three reviewers (YW, YZH, and WCH) screened articles according to inclusion and exclusion criteria. Three reviewers independently reviewed the studies' title and abstract, then screened the full paper, and independently evaluated methodological quality. Any uncertainties concerning suitability were discussed at weekly group meetings with all reviewers.

#### Data Extraction

Three reviewers independently extracted data using a predesigned form from the included studies. The following data were extracted: (1) basic study information, namely, authors, reference, and country/region; (2) participant characteristics, namely, illness/condition, total number, and number in each group, age, gender, and race/ethnicity; (3) intervention characteristics, namely, intervention content, individual or group format, in-person or virtual, setting, length (e.g., number of weeks), number of sessions, duration per session, and control; (4) intervention assessment information, namely, time point (e.g., pretest, posttest, follow-up), measures, outcomes with screenshots (including the mean, standard deviation, and number of participants in each group at each time point), and outcome raters (e.g., patients, caregivers, and staff); and (5) information on bias risk assessment (see *Assessment of risk of bias in included studies*). After comparing results, any uncertainties that could not be solved were discussed in weekly meetings with all reviewers.

#### Data Analysis

RevMan 5.4 was used for meta-analysis. Meta-analysis was performed if outcomes were measured by the same scales in at least two studies. Heterogeneity was assessed using an *I*^2^ statistic. To interpret heterogeneity, reviewers followed Cochrane guidance: 0–40% as not important, 30–60% as moderate heterogeneity, 50–90% as substantial heterogeneity, and 75–100% as considerable heterogeneity (37). A random-effects model was used if I^2^ statistics reports the value of 50% or above. A fixed-effects model was used if *I*^2^ statistics were lower than 50%. If one study used more than one instrument to measure the same outcome variable, the team employed the more commonly used instrument for the analysis.

Subgroup analyses were conducted with the following characteristics if applicable: outcome instrument, disease/conditions, country/region, rater, and follow-up. If the heterogeneity showed moderate or high, we performed subgroup and sensitivity analysis. Publication biases were assessed by a funnel plot if the number of studies used for meta-analysis was more than 10.

## Results

### Search Results

**Figure 1:**
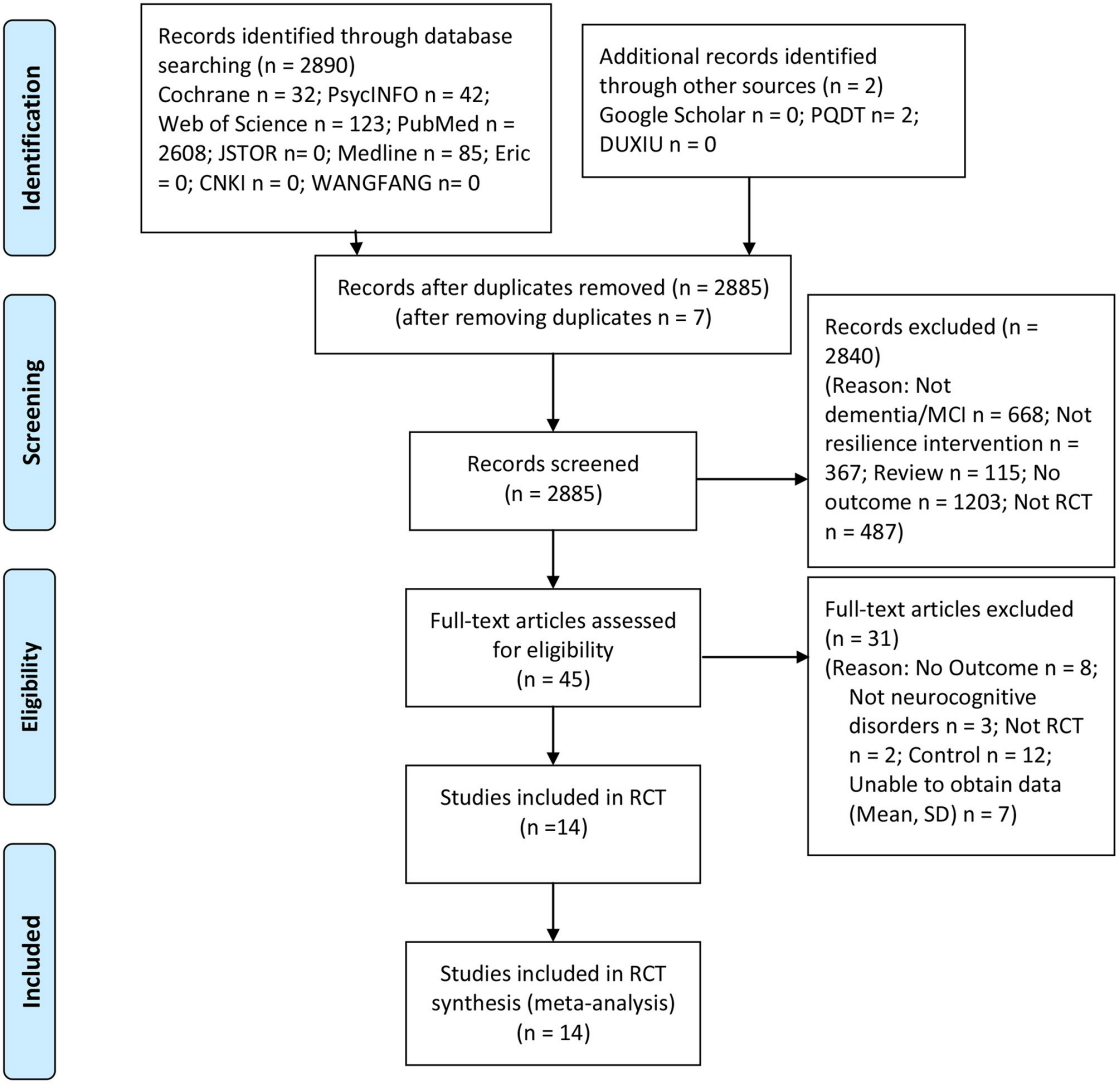
Flowchart of the study selection process.

Figure 1 shows the Preferred Reporting Items for Systematic Reviews and Meta-analyses (PRISMA) flow chart of the study review and selection process. A total of 2,890 studies were searched from electronic searches. After deduplication, we considered a total of 2,885 studies. The remaining studies were screened at the title and abstract level based on the pre-established inclusion and exclusion criteria, depending on the study type, population, intervention, control, and outcome. Forty-five full papers were then reviewed, from which 31 were excluded utilizing the same criteria. Fourteen RCT studies satisfied all the inclusion criteria. Thus, 14 RCT studies representing a total of 2,442 participants with neurocognitive disorders were included in the systematic review and meta-analysis.

### Included Study Characteristics

#### Location

Among 14 studies, 7 were conducted in the UK, 2 in the USA, 1 in Germany, 1 in Denmark, 1 in France, 1 in Netherlands, and 1 in Norway (Table 1).

**Table 1:** Characteristics of included studies.

**Study Reference**	**Participants**	**Study Design**	**Intervention Group (IG)**	**Control Group (CG)**	**Outcome Measures**	**Data Collection Time and Raters**	**Results**
Luttenberge et al. (38)	*N* = 139 (119 were analyzed); Mean age = 85 y/o; Residents with degenerative dementia from five German nursing homes	RCT	*N* = 71 (56 were analyzed) 6 months intervention comprised three segments: activities of daily living, motor stimulation, and cognitive stimulation (MAKS). Ten patients under the guidance of two therapists participated in the standardized intervention for 2 h, 6 days a week	*N* = 68 (63 were analyzed)Controls received treatment as usual	NOSGER (subscale: Mood, Social behavior, ADLs, IADLs)	Pre, post (6 months); Rated by nursing home staff	This effect was greatest on the social behavior (*p* < 0.01) and instrumental activity of daily living (IADLs) (*p* = 0.01)
Samus et al. (39)	*N* = 303 (265 with dementia, 38 with mild cognitive impairment; age ≥70 y/o, community-living, in Baltimore, MD, USA	RCT	*N =* 110 Care coordination intervention to systematically identify and address dementia-related caregiving according to people-centered caregiving planning; dementia education and skill-building strategies caregiving monitoring by an interdisciplinary team. Weekly in-person 2-h meetings	*N =* 193Usual care	1. QOL-AD-participant 2. QOL-AD-proxy 3. ADRQL-40 4. NPI-Q 5. CSDD	Pre, post, f/u (9 months, 18 months); self-reported participants and masked evaluators	1. IG had a significant improvement in self-reported QOL 2. No group differences were found in neuropsychiatric symptoms, depression, or proxy-rated QOL QOL-AD-Self (18 months, *p* = 0.027); ADRQL-40 (18 months *p =* 0.568); QOL-AD-Informal (18 months, *p =* 0.592); CSDD (18 months *p =* 0.925); NPI-Q-Severity (18 months, *p =* 0.233)
Saint-Bryant et al. (26)	1. Dementia, *N =* 19; Mean age = 88 y/o; mild or moderate dementia 2. Staff participants, *N =* 21 Participants from care homes in the UK	RCT	*N =* 10 1.The SettleIN program: staff-led; four required interventions: orientation, friends and family, identity, and lifestyle, and an optional intervention for residents that have difficulty engaging. Content included a range of activities designed to support the healthy adjustment 2. Four weeks for a full-time staff to complete, with a maximum of 6 weeks for part-time staff. Lasting 1 h and 15 min	*N =* 9residential care as usual	1. CSDD 2. QOL-AD 3. IRA	Pre, post (week seven); Rated by staff participants	There was no significant difference regarding QOL, psychological well-being, or overall adjustment outcomes CSDD *p* = 0.17; QOL-AD *p* = 0.43; IRA *p* = 0.24
Waldorff et al. (40)	*N =* 330 Age ≥ 50 y/o, carers, and individuals with Alzheimer's disease, living in a nursing home, Denmark	RCT	*N =* 167, mean age: IG = 85.2, CG = 85.9); DAISY intervention: multifaceted and semitailored education, counseling, and support. All courses lasted 2 h; telephone approximately five to eight times every 3–4 weeks	*N =* 163Routine follow-up	1. QOL-AD 2. NPI-Q 3. CDS (Cornell depression scale)	Pre, post, f/u (6 months, 12 month); Rated by trained raters	1. QOL-AD and NPI-Q outcome did not have any significant effect at 12 months 2. Non-significant but a small difference was observed for CDS in favor of IG (*p* = 0.0146 and *p* = 0.0103, respectively)
Surr et al. (41)	*N =* 726 (intervention group: mean age = 86 y/o, controls mean age = 85 y/o); permanent resident of the care home, officially diagnosed with dementia, in care homes in the UK	RCT	*N =* 418 dementia, *N =* 31 care homes Adhered to standard procedures specified in the DCM Dementia Care Mapping™) manual and guidelines in the provision of individualized person-centered care including five components, such as observation, reporting, feedback, and action planning; 38 min a time, session 1–2 weeks	*N =* 308 dementia*N =* 19 care homes, usual care	1. CMAI 2. NPI 3. QOL-AD 4. PAS	Pre, post, f/u (6 months,16 months); Rated by independent researchers	1. CMAI was lower in IG than in CG (*p =* 0.104 2. NPI, PAS, and QOL-AD outcomes were not clinically effective at reducing agitation or improving QOL and other outcomes
Jha et al. (27)	*N =* 48 (34 people completed the trial) people with MCI and early dementia and their family carers from villages in Hertfordshire in the UK Age ≥ 65 y/o	RCT	*N =* 17 Recovery-orientated psychiatric therapeutic: three components: (a) prediagnostic counseling and well-being assessment; (b) therapeutic diagnostic consultation; (c) written feedback. lasting at least an hour for 6 months	*N =* 17Control group (treatment, as usual, TAU)	1. WHO-5 2. CSDD 3. EQ-5D 4. ZBI	Pre, post (6 months); Rated by the clinician	IG exhibited a significant enhancement in the WHO Well-being Index (*p =* 0.03). There were also trends of improvement in other outcome measures WHO-5 (*p =* 0.03) CSDD (*p =* 0.38) EQ-5D (*p =* 0.66) ZBI (*p =* 0.90)
Vickrey et al. (42)	*N =* 408 dyads 408 patients with dementia age ≥65 y/o paired with 408 informal caregivers. Three health care organizations in collaboration with 3 community agencies in southern California, USA	RCT	*N =* 238 dyads 1. A dementia guideline-based disease management program for more than 12 months 2. Dementia care managers (mostly social workers) employed a web-based care management software system for care planning and coordination 3. The care management protocol included ongoing follow-up, usually via phone, with a needs-based frequency and a formal reassessment every 6 months to assess the need for major revisions to the caregiving plan 4.At each intervention clinic, over 90 min of standardized, interactive seminars (in up to 5 sessions)	*N =* 170 dyads Usual Care	1. Receipt of community resources 2. HUI3	Pre, post, f/u (12–18months); Rated by caregivers	1. Higher proportions received community agency assistance (*p =* 0.03) than those who received usual care 2. Patient health-related QOL, caregiving quality, overall quality of patient care, level of unmet caregiving assistance needs, and social support were better for IG than CG (*p =* 0.05)
Dechamps (43)	*N =* 160 Age: 65–102 y/o, mean *=* 82.3 Neuropsychiatric diagnosis as dementia and psychosis; from the long-term care home and the nursing homes, France	RCT	Exercise regimen/activity program 1. Adapted Tai Chi (AT): *N =* 51 Exercise program: 4 sessions of 30 min a week for 6 months 2. the Cognition-Action program (CA): *N =* 49 CA is a training program to enhance adhesion to exercise by adding a meaning to exercise: 30 min and advanced to 40 min twice a week for 6 months	*N =* 60Usual care	1. GDS 2. NPI 3. ADL	Pre, post, f/u (6 and 12 months); Rated by researchers	1. ADL score has no significance (AT: *p =* 0.24 and CA: *p =* 0.15) 2. NPI was unchanged or improved in the intervention groups (*p* > 0.001) 3. Neuropsychiatric diagnosis subgroups did not respond to any interventions
Lowery et al. (44)	*N =* 131 dyads 1. Control mean age = 78 y/o 2. Intervention mean age = 79 y/o 3. Participants with dementia living home from Diseases Research Network's dementia research register from the North Thames, in the UK	RCT	Exercise regimen/activity program *N =* 64 Exercise regimen in addition to TAU (treatment) Customized walking regimen designed to gradually become intensive and last from 20 to 30 min, at least five times a week	*N =* 67Treatment as usual	1. DemQOL-Proxy 2. NPI	Pre, post, f/u (6, 12 weeks); Rated by the research worker	1. No significant difference was found between the groups of mean NPI (6 weeks: *p =* 0.76;12 weeks: *p =* 0.6) 2. No statistically significant differences between the groups in QOL of participants with dementia (measured using the Demqol-proxy) (6 weeks: *p =* 0.49;12 weeks: *p* = 0.09)
Henskens et al. (28)	*N =* 87 Age ≥65 y/o 87 residents with dementia living in a psychogeriatric ward of nursing home (NH), Amsterdam, the Netherlands	RCT	Exercise regimen/activity program *N =* 43 ADL training, a multicomponent aerobic and strength exercise training, and combined ADL and exercise training. Receive three 3-h educational sessions. strength and aerobic exercises, with a frequency of three times a week, 30–45 min each session. Sessions rotated weekly, consisting of two strength sessions and one aerobic session, followed by a week of two aerobic sessions and one strength session. Subgroups: 1. the exercise (physical activity) PADL; 2. the exercise (physical activity) PCO	*N =* 44Care-as-usualSubgroups: 1. Social activity SADL2. Social activity SCO	1. CDS (the Care Dependency Scale) 2. E-ADL 2. Qualidem (subscale: care relationship; positive affect; negative affect; restless tense behavior; positive self-image; social relations; social isolation; feeling at home; having something to do	Pre, post, f/u (3 and 6 months); rated by physiotherapists	1.The ADL training positively affected overall QOL (*p =* 0.004) and its multiple aspects: care relationship (*p =* 0.004), positive self-image (*p =* 0.002), and feeling at home (*p =* 0.001) compared to care-as-usual 2. No benefits were observed of exercise on QOL 3. No benefits were observed from a combined ADL and exercise interventions on QOL 4. No effects were found of the three-movement interventions on ADL performance
Jøranson et al. (45)	*N =* 60, age range 62–95 y/o residents with dementia or cognitive impairment in 10 nursing home units, Norway	RCT	Exercise regimen/activity program *N =* 27 A robot-aided group activity with the robot seal Paro Group sessions in a separate room for 30 min twice a week for 12 weeks	*N =* 26treatment as usual	1. BARS 2. CSDD	Pre, post, f/u (12 weeks, 3 months)Rated by staff	There were statistically significant differences in changes in agitation and depression between groups from T0 to T2. No significant differences in changes on agitation or depression between groups from T0 to T1 T1–T0: BARS: *p* = 0.098; CSDD: *p =* 0.098 T2–T0: BARS: *p =* 0.048; CSDD: *p =* 0.028
Churcher Clarke et al. (29)	*N =* 31, age range 61–95 y/o; people with dementia; From care homes in the UK	RCT	*N =* 20 A group-based adapted mindfulness intervention plus treatment as usual: a 10-session intervention, comprising 10, 1-h group sessions, running twice per week for 5 weeks at the care home	*N =* 11Treatment as usual	1. CSDD 2. RAID 3. QOL-AD 4. PSS-13	Pre, post (1week postintervention)Rated by patients	There was a significant improvement in QOL in IG compared to CG (*p =* 0.05). No significant changes in other outcomes
Woods et al. (46)	*N =* 488 Mean age = 77.5 y/o individuals with mild to moderate dementia living in family homes in the UK	RCT	*N =* 268 Joint reminiscence, 12 consecutive weeks + monthly maintenance sessions for an additional 7 months. Twelve 2-h weekly sessions	*N =* 219Usual care	QOL-AD	Pre, post, f/u (3 months, 10 months); self-reported	No differences in outcome between the IG and CG at the 10-month endpoint (*p* = 0.53)
Ali et al. (47)	*N =* 40 dyads Carer and individual with dementia from communities Age ≥40 y/o, in the UK	RCT	*N =* 20 dyads 1. Dementia individual cognitive stimulation therapy (iCST arm) and treatment as usual 2. 40 sessions over 20 weeks: warm-up, orientation, the main activity) twice a week for 30 min per session	*N =* 20 dyadsA waiting list control group received treatment as usual	QOL-AD	Baseline, midpoint (week 11), the end (week 21)Rated by the research assistant	QOL was significantly higher in the iCST arm at 21 weeks (week 11 *p =* 0.61; week 21 *p =* 0.02)

*QOL-AD, Quality of Life–Alzheimer Disease scale; QOL-AD-participant, Quality of Life in AD, which was administered to participants; QOL-AD-proxy, Quality of Life in AD for study partners; DemQOL-Proxy, quality of life of participants with dementia; DQOL, Dementia Quality of Life instrument.; ADRQL-40, the Alzheimer's Disease Rated Quality of Life-40 item scale; EQ-5D, EUROQOL; WHO-5, WHO Well-Being Index; GAS, Goal Attainment Scale; NOSGER, the Nurses' Observation Scale for Geriatric Patients (subscale including: Mood, Social behavior, ADLS, IADL); Qualidem assesses QOL and subscales including (1) care relationship, (2) positive affect, (3) negative affect, (4) restless tense behavior, (5) positive self-image, (6) social relations, (7) social isolation, (8) feeling at home, (9) having something to do; GDS, Geriatric Depression Scale; CDS, Cornell depression scale; CSDD, the Cornell Scale for Depression in Dementia; CS, Cornell scale for depression in dementia; ABID, Agitated Behaviors in Dementia scale; BARS, the Brief Agitation Rating Scale; CMAI, Cohen-Mansfield Agitation Inventory; PAS, the Pittsburgh Agitation Scale; RAID, the Rating Anxiety in Dementia Scale; NPI, Neuropsychiatric Inventory; NPI-Q, the Neuropsychiatric Inventory questionnaire; IRA, the Index of Relocation Adjustment; PSS-13, Perceived Stress Scale; ZBI, Zarit Burden Interview; CDS, Care Dependency Scale; HUI3, the Health Utilities Index Mark 3; IADL, instrumental activities of daily living; ADL, activities of daily living; E-ADL, Assessors of the Erlangen ADL*.

#### Participants

Participants in most studies (*n* = 10) were persons with dementia (26, 28, 29, 38, 41–44, 46, 47), one as persons with MCI (39), one as persons with MCI and early dementia (27), one as persons with dementia or cognitive impairment (45), and one was Alzheimer's (40). The number of participants ranged from 19 to 726, and seven studies had more than 100 participants. Participants were recruited from communities (*n* = 6) and nursing homes/residential care facilities (*n* = 8).

#### Interventions

We identified six resilience approaches based on content descriptions, including integrated approaches (*n* = 5), exercise regimen or activity programs (*n* = 4), psychological interventional technique (*n* = 2), a psychiatric intervention (*n* = 1), disease/case management (*n* = 1), and cognitive stimulation therapy (*n* = 1). Among five studies with integrated approaches, one was individualized person-centered care provided by standard procedures to diminish turmoil in care home residents with dementia (41), one was multimodal non-drug therapy on dementia's symptom and care need (38), one was multidimensional home-based care coordination provided by an interdisciplinary team to maximize independence for persons with MCI living home (39), one was a staff-led intervention that comprises four mandatory modules and one optional module to facilitate the adaptation of seniors with dementia after placement into residential care (26), and one was a psychosocial intervention including multifaceted and semi-tailored counseling, education, and support (40).

Four studies provided exercise regimens or activity programs (28, 43–45), such as adapted Tai Chi and cognition-action program, walking, aerobic, and strength exercise training. Two mental interventional techniques used mindfulness (29) and reminiscence (46). One study of recovery-orientated psychiatric intervention packages included prediagnostic well-being assessment and counseling, diagnostic consultation with written feedback, and postdiagnostic support (27). One disease management employed an internet-based care management software system for care planning and coordination (42). One individual cognitive stimulation therapy intervened and assessed adaptive functioning and QOL of participants with dementia (47). All interventions were conducted in groups of persons older than 40 years old.

Regarding intervention intensity, the length ranged from 6 weeks to 12 months. The duration of sessions included 30 min twice a week (*n* = 2), 40 min twice a week (*n* = 1), 30–45 min once a week (*n* = 1), 38 min once per 1–2 weeks (*n* = 1), 20–30 min at least five times per week (*n* = 1), 1 h at least once a week (*n* = 4), and 2 h, 6 days a week (*n* = 4).

#### Comparators

Control groups included usual care or TAU (*n* = 12), routine follow-up (*n* = 1) (40), and a wait-list control group (*n* = 1) (47).

#### Outcome Measurement

Table 1 shows that 14 studies assessed psychosocial outcomes, including QOL, well-being, mood state, neuropsychiatric symptom, positive self-image, adaption, goal attainment, and adjustment. Most studies evaluated QOL (*n* = 11). Specifically, QOL was rated by seven scales [Quality of Life in Alzheimer Disease (QOL-AD-participant), Quality of Life in A.D. for study partners (QOL-AD-proxy), quality of life of participants with dementia (DemQOL-Proxy), Dementia Quality of Life instrument (DQOL), the Alzheimer's Disease Rated Quality of Life-40 item scale (ADRQL-40), EUROQOL (EQ-5D), the Health Utilities Index Mark 3 (HUI3), and QOL-AD] in 11 studies. Mental well-being was rated by one scale [WHO Well-being Index (WHO-5)] in one study. Adaption was rated by three scales [Perceived Stress Scale (PSS-13), Zarit Burden Interview (ZBI), and Cornell depression scale (CDS)] in three studies. Goal attainment was rated by one scale [Goal Attainment Scale (GAS)] in one study. Adjustment was rated by one scale (IRA) in one study. Mood state was assessed by 11 scales, including depression [Geriatric Depression Scale (GDS), Cornell Scale for Depression in Dementia (CS), CDS, and CSDD] in seven studies, agitation [Agitated Behaviors in Dementia scale (ABID), the Brief Agitation Rating Scale (BARS), Cohen–Mansfield Agitation Inventory (CMAI), and the Pittsburgh Agitation Scale (PAS)] in three studies, and anxiety [the Rating Anxiety in Dementia Scale (RAID)] in one study. Neuropsychiatric symptom was rated by two scales [Neuropsychiatric Inventory (NPI) and the Neuropsychiatric Inventory questionnaire (NPI-Q)] in five studies. In addition, resilience was rated in two studies by two comprehensive scales [Qualidem and the Nurses' Observation Scale for Geriatric Patients (NOSGER)], including positive self-image, social relations, and mood.

All 14 studies had pre- and postintervention assessments. Eleven studies had follow-up (f/u) assessments, and the f/u assessments were conducted at different time points (e.g., 7, 12, and 21 weeks and 3, 6, 10, 12, 16, and 18 months).

#### Effects of Interventions

Overall, the effects of resilience interventions were diverse in various outcomes. In terms of social behavior, one study indicated significant differences in favor of resilience interventions compared with controls (38). Meanwhile, no significant differences were found in neuropsychiatric symptoms (39–41, 43, 44), adjustment (26), stress (29), and anxiety (29) in favor of resilience interventions compared with controls. Besides, the results of other outcomes' assessments were inconsistent. Specifically, regarding QOL, seven studies showed no statistical significance in favor of resilience interventions compared with controls (26, 27, 40–42, 44, 46), while two showed a significant effect (29, 47). One showed a significant enhancement in self-reported QOL but no significant improvement in proxy-rated QOL (39). One showed that activities of daily living (ADL) training had positively affected overall QOL, but no benefits were observed for exercise on QOL (28). As for depression, four included studies showed that the resilience interventions were not statistically different compared with controls (27, 29, 39, 40), while one showed statistically significant differences (45). In terms of well-being, one study showed no significance in favor of resilience interventions compared with controls (26), while the other study showed statistical significance (27). About the effects on agitation, one showed no significance in favor of resilience interventions compared with controls (41), while one showed statistical significance (45).

### Risk of Bias

**Figure 2:**
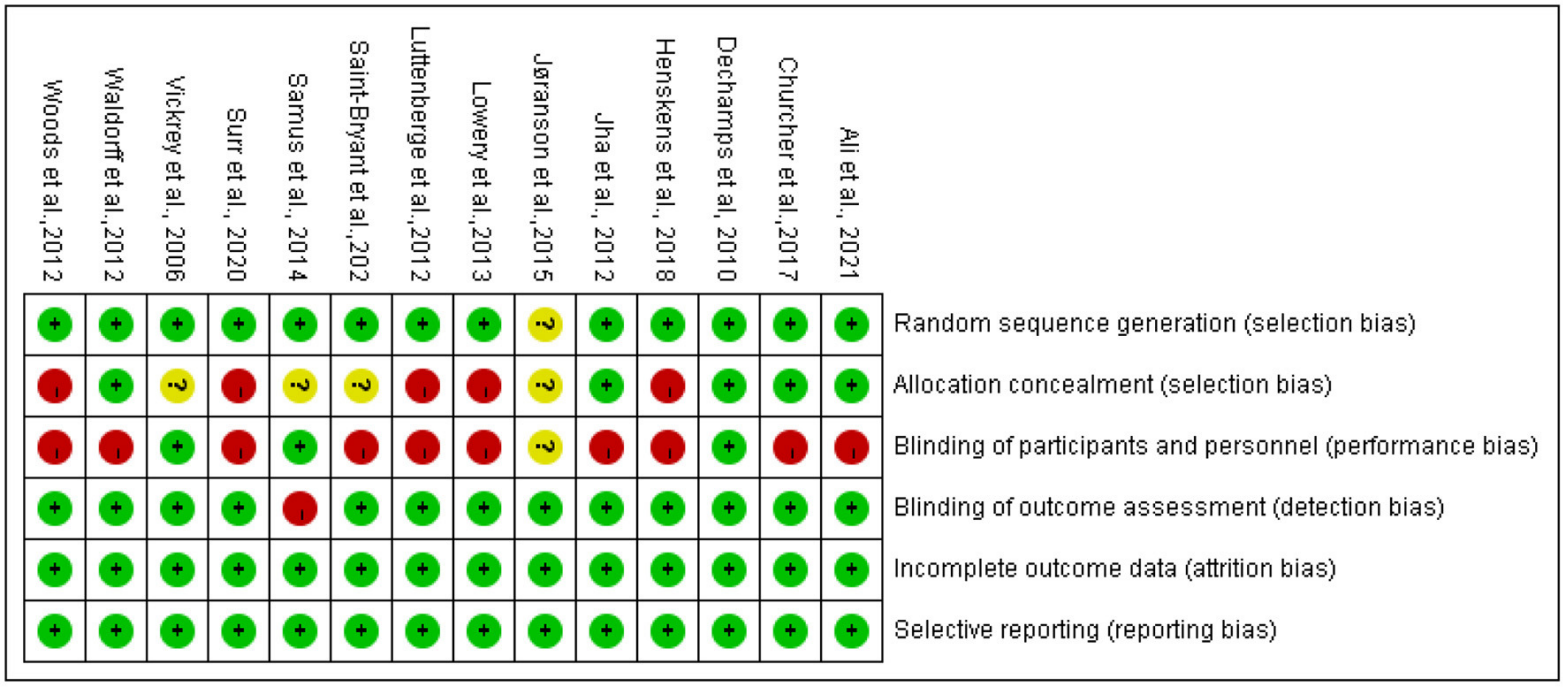
Risk of bias summary.

Risks of bias are summarized in Figure 2. The main flaws for risks of bias across 14 studies were in allocation concealment and blinding participants and interventionists. Regarding random sequence generation, 13 studies were judged to be at low risk, which used computer-generated random numbers, a block randomization method, a custom Excel program, or a web-based system.

For allocation concealment, five studies were rated as low risk, which used sealed envelopes, allocation numbers by a blind assigner, or emphasis on allocation concealment. Five studies were judged to be high risk, which reported no concealment in the allocation process. The remaining four studies did not report information on allocation concealment and were rated as unclear.

Regarding blinding participants and interventionists, 3 studies were rated as low risk, 10 studies as high risk, and 1 study as unclear. Meanwhile, most studies (*n* = 13) were judged as low risk for blinding outcome assessment, by using personnel not included in the intervention process, and one study was judged as high risk due to non-blinding outcome assessment.

All studies were judged to be at low risk for incomplete outcome data because the dropout rate was low (<30%) during the intervention, and they explained the numbers and reasons for dropout and the data analysis methods of dealing with missing values. Lastly, all studies were judged as low risk for selective reporting.

### Meta-Analysis Results for Quality of Life

#### Meta-Analysis

**Figure 3:**
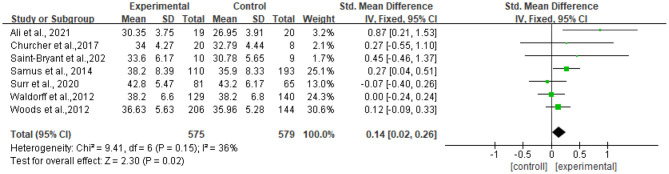
Effect size of the intervention group vs. the control group on QOL-AD rating scores.

Seven studies reported data on quality of life, assessed by QOL-AD and were pooled for a meta-analysis using a fixed-effects model. Results illustrated that there were significant standardized mean differences in favor of resilience interventions compared with controls for QOL [SMD = 0.14, 95% CI (0.02, 0.32), *p* = 0.02] (Figure 3).

#### Subgroup Analyses

**Figure 4:**
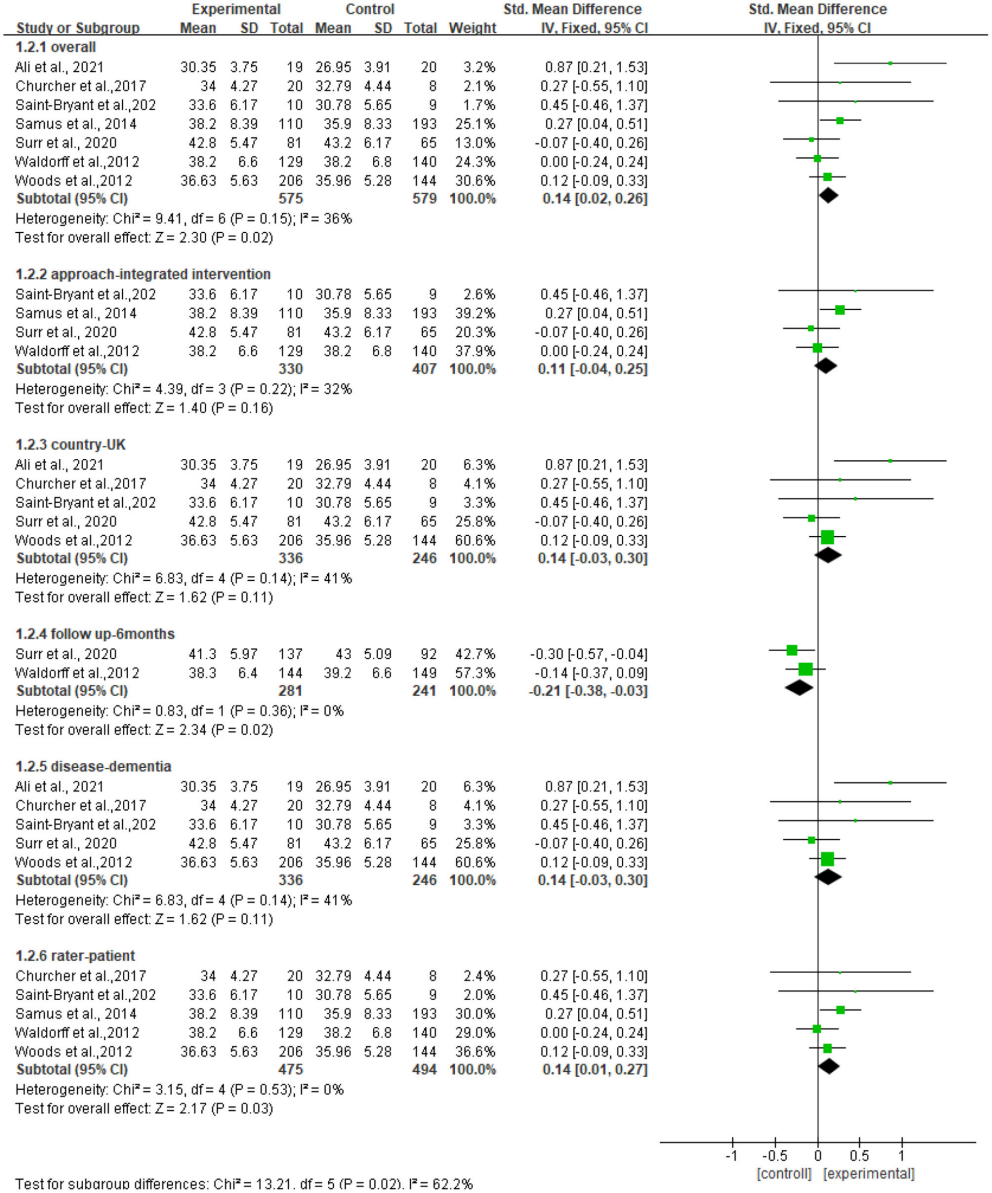
Subgroup analyses of the intervention group vs. the control group on QOL-AD rating scores.

We further performed subgroup analyses with results shown in Figure 4. The subgroup result of the studies of the f/u assessments with 6 months showed significant standardized mean differences in favor of controls compared with resilience interventions [SMD = −0.21, 95% CI (−0.38, −0.03), *p* = 0.02] and no heterogeneity (*I*^2^ = 0%). The subgroup result of the studies of outcome rated by patients showed significant standardized mean differences in favor of resilience interventions compared with controls [SMD = 0.14, 95% CI (0.01, 0.27), *p* = 0.03] and no heterogeneity (*I*^2^ = 0%). Other subgroup result analyses showed no significant standardized mean differences in favor of resilience interventions compared with controls, and moderate heterogeneity (*I*^2^ = 36%−41%), which included the subgroup of the studies of persons with dementia [SMD = 0.14, 95% CI (−0.03, 0.30), *p* = 0.11], the subgroup of the studies of approaches using integrated approaches [SMD = 0.11, 95% CI (−0.04, 0.25), *p* = 0.16], and the subgroup of the studies conducted in the UK [SMD = 0.14, 95% CI (−0.03, 0.30), *p* = 0.11].

#### Assessment of Sensitivity

**Figure 5:**
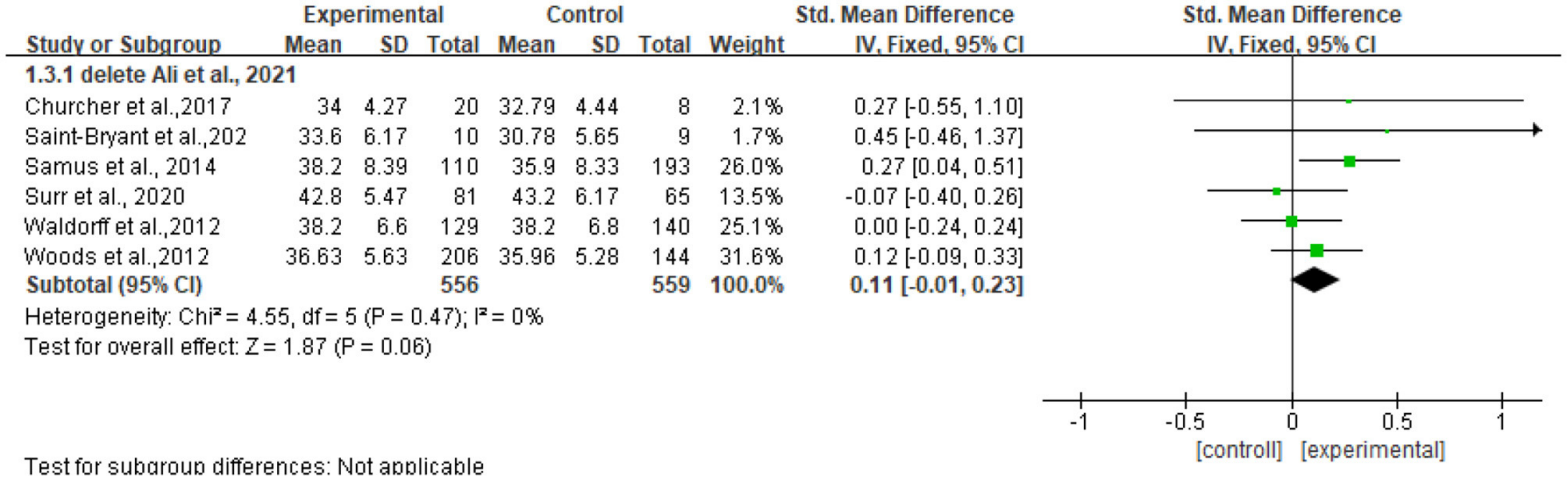
Sensitivity analyses of the intervention group vs. the control group on QOL-AD rating scores.

The heterogeneity of the seven included studies was moderate (*I*^2^ = 36, chi^2^ = 9.41), which suggested that heterogeneity might not be important as explained in Cochrane guidance. The sensitivity analysis showed no heterogeneity (*I*^2^ = 0% chi^2^ = 4.55) after a small sample study (*n* = 40) (47) was deleted (see Figure 5).

#### Analysis of Publication

Only seven RCTs were included, so the funnel plot was not made, but publication bias may exist.

### Meta-Analysis Results for Depression

#### Meta-Analysis

**Figure 6:**
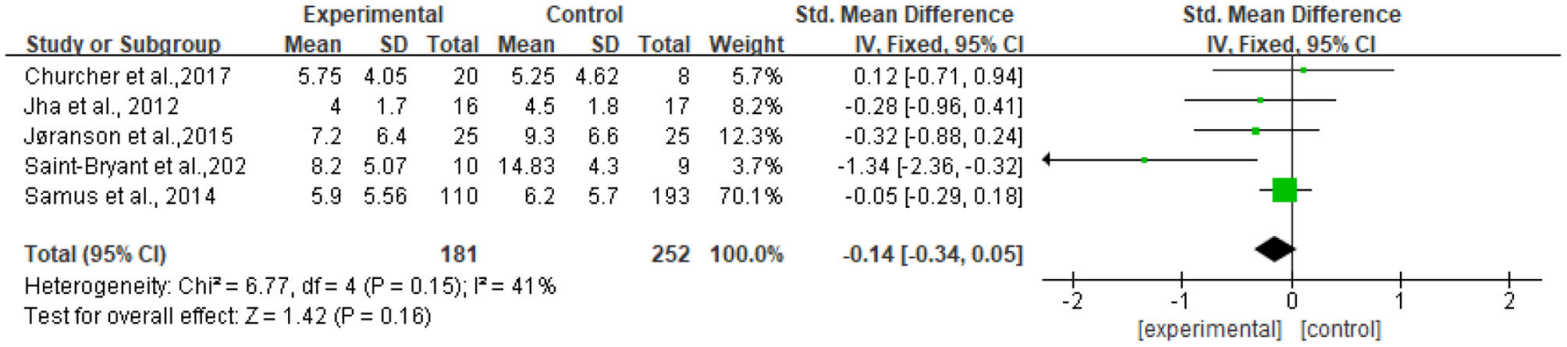
Effect size of the intervention group vs. the control group on CSDD.

Five studies reported data on depression assessed by the CSDD and were pooled for a meta-analysis using a fixed-effects model. Results demonstrated that there were no significant standardized mean differences in favor of resilience interventions compared with controls for depression [SMD = −0.14, 95% CI (−0.34, 0.05), *p* = 0.16] (Figure 6).

#### Subgroup Analyses

**Figure 7:**
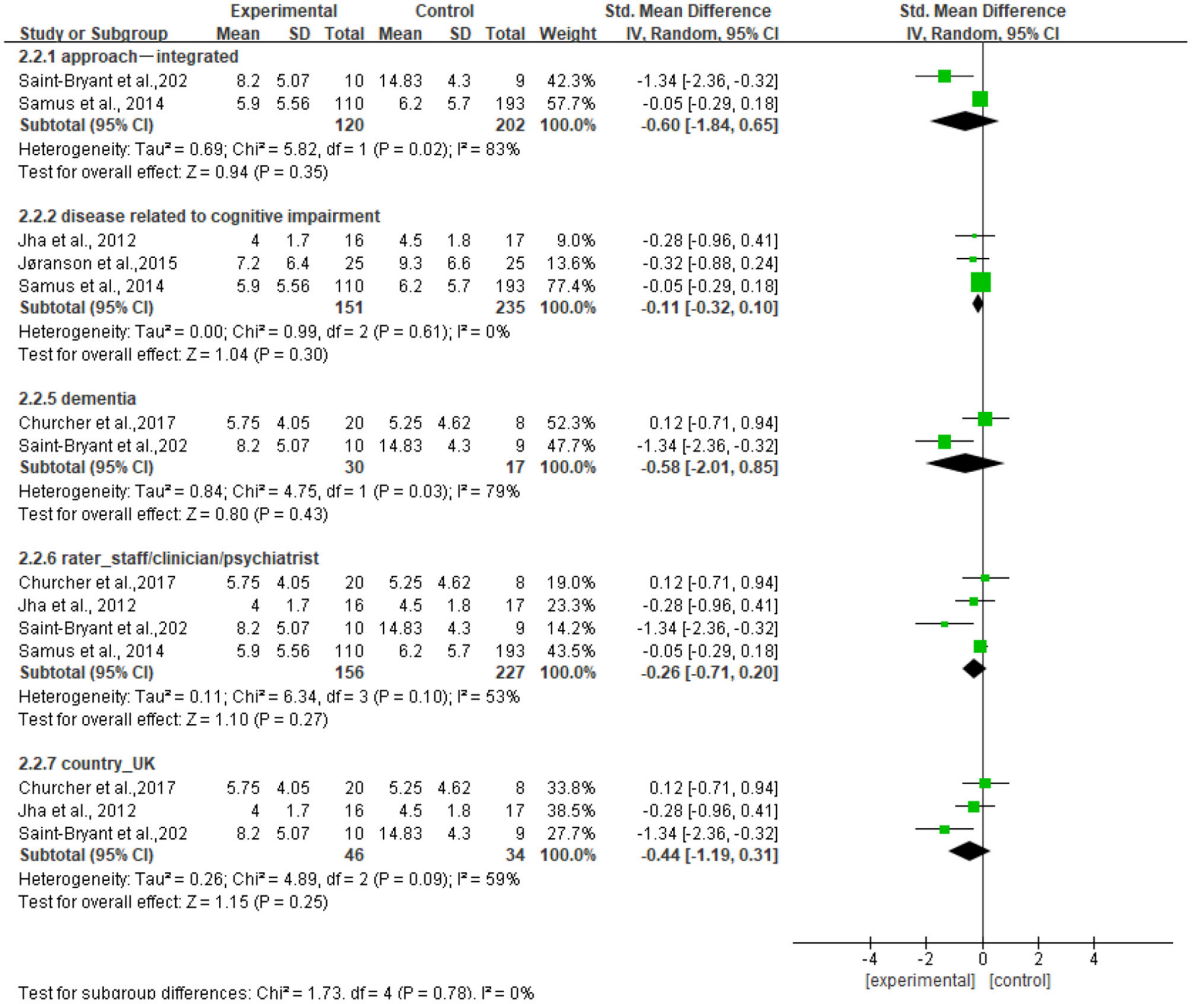
Subgroup analyses of the intervention group vs. the control group on CSDD.

Figure 7 shows that no significant standardized mean differences in favor of resilience interventions compared with controls for CSDD were found in subgroup analyses. The heterogeneity ranged from 0 to 83%. Specifically, the subgroup result of participants with dementia showed no heterogeneity (*I*^2^ = 0%). Other subgroup results showed moderate or high heterogeneity (*I*^2^ = 53%−83%).

#### Assessment of Sensitivity

**Figure 8:**
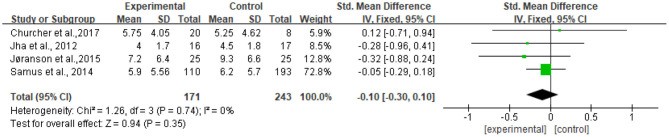
Sensitivity analyses of the intervention group vs. the control group on CSDD rating scores.

The heterogeneity of the five included studies was moderate (*I*^2^ = 41 chi^2^ = 6.77). The sensitivity analysis showed that the heterogeneity decreased (*I*^2^ = 0% chi^2^ = 1.26) after a small sample study (*n* = 19) (26) was deleted (see Figure 8).

#### Analysis of Publication Bias

Only five RCTs were included, so the funnel plot was not made, but publication bias may exist.

### Meta-Analysis Results for Neuropsychiatric Symptoms

#### Meta-Analysis

**Figure 9:**
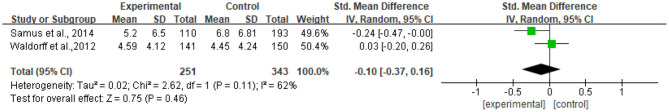
Effect size of the intervention group vs. the control group on NPI-Q.

Two studies reported data on neuropsychiatric symptoms assessed by NPI-Q and were pooled for a meta-analysis using the random-effects model. Results revealed that there were no significant standardized mean differences in favor of resilience interventions compared with controls for neuropsychiatric symptoms [SMD = −0.10, 95% CI (−0.37, −0.16), *p* = 0.46] (Figure 9). Meanwhile, there tended to be substantial heterogeneity in the two included studies (*I*^2^ = 62%, chi^2^ = 2.62).

#### Analysis of Publication Bias

Only two RCTs were included, so the funnel plot was not made, but publication bias may exist.

## Discussion

This systematic review and meta-analysis examined the effectiveness of resilience interventions among persons with neurocognitive disorders. A total of 14 RCT studies representing 2,442 participants were identified that fulfilled the inclusion criteria of this review. The risk of bias was either high or unclear for most studies in allocation concealment, blinding participants, and interventionists domains. Meta-analyses were conducted for a primary outcome of QOL and secondary outcomes of depression and neuropsychiatric symptoms. Our results indicated that resilience interventions had a significant positive effect on persons with neurocognitive disorders in enhancing QOL but might not be beneficial in decreasing depression and neuropsychiatric symptoms. Meanwhile, many other psychosocial outcomes were measured less frequently.

Our review identified target groups of neurocognitive disorders based on various conditions including symptoms and level of severities: mild or moderate dementia, cognitive impairment or MCI, and Alzheimer's disease. Similarly, specific target groups in the review of Regan and Varanelli (33) included mild cognitive impairment and early dementia. Inconsistent with our study, some other reviews (31, 32) focused on mixed target groups, including both healthy older adults and older adults at risk of dementia, MCI, and Alzheimer's disease, focusing on the early prevention and intervention of neurocognitive disorders. This might also be one reason why it was impossible to judge the effect of interventions due to sample heterogeneity.

Meanwhile, our review included RCTs and conducted meta-analyses to assess the effect of resilience interventions. Consistent with our study, two previous systematic reviews also contained RCTs, which included 12 RCTs (31) and 47 RCTs (32), respectively. However, they applied a narrative approach to synthesize the findings without conducting a meta-analysis. Inconsistent with our study, the review of Regan et al. (33) included other study designs, such as pre–post studies, besides RCTs, which might be one of the reasons why the findings were inconclusive. However, the quality of RCTs included in our review is not very high. Only 5 of the 14 studies were judged low risk in allocation concealment, and only 3 of the 14 studies were judged low risk in blinding of participants and interventionists domains. Although it is very difficult to implement allocation concealment and blinding of participants and interventionists domains in real-world RCT research, it is strongly recommended that more rigorous RCT research should be carried out in the future, with special attention to allocation concealment and blinding of participants and interventionists domains.

In addition, our review identified integrated resilience approaches. In contrast, the reviews of Regan and Varanelli (33) focused on psychotherapeutic approaches; the review of Li et al. (31) paid more attention to resistance training, strength, and exercise programs; and the review of Carrion et al. (32) solely focused on cognitive therapy. A broader range of resilience approaches was considered in our review, which rendered interventions diverse. Among them, integrated resilience approaches that were used by most included studies involved multiple components, such as caregiver's support, social connection, and resource support. Results indicated the possible advantages of the multiple-component interventions and multidisciplinary teamwork in active coping with complex symptoms and stress of persons with neurocognitive disorders. Thus, we call for more resilience research using integrated approaches for persons with dementia to better understand the effectiveness of integrated approaches and how to appropriately adopt and implement them.

Furthermore, our review focused on psychosocial outcomes (e.g., QOL, ADL, mental health, coping ability, adaption, adjustment). However, social outcomes were measured less frequently. The meta-analysis of psychological outcomes was conducted, so it is impossible to judge the effect of resilience interventions on social outcomes, such as improving social connection, social well-being, and resource support. Similarly, Regan and Varanelli (33) assessed depression, anxiety, and adjustment. Carrion et al. (32) rated depression. In contrast, Li et al. (31) largely focused on the effect of resistance training on cognitive function, such as executive cognitive ability, global cognitive function, attention, and memory. Since there is no “gold standard” for measuring the outcomes of resilience interventions in persons with neurocognitive disorders, we recommend that the measurement instruments can be further developed and validated to measure more effectively.

Additionally, regarding the effectiveness of resilience interventions, the previous systematic reviews' findings indicated positive effects (31, 33) or inconclusive (32) on different outcomes. However, due to the different design of the included original studies or the lack of meta-analysis of RCTs in these reviews, the statistical significance of the effect of resilience interventions could not be judged, and the level of evidence was not high. Therefore, these conclusions of intervention effects needed to be drawn cautiously. One synthesis of systematic reviews also indicated that due to the heterogeneity of the included studies, there was no sufficient evidence to determine whether resilience interventions may promote psychosocial outcomes (48). Our meta-analysis of the RCTs confirmed that resilience interventions had significant benefit to persons with neurocognitive disorders in enhancing QOL but might not be beneficial in decreasing depression and neuropsychiatric symptoms. It should be pointed out that several aspects of the original study limited the generalizability of our results: distinct approaches, diverse measurement tools and raters, and divergent settings and locations, and different data collection points during interventions and in f/u assessments. Therefore, further research is needed to address the development, implementation, and application of resilience interventions and conduct more rigorous and higher-quality RCT trials among persons with neurocognitive disorders.

This study has several limitations. First, due to the research team's language capacity, we only included English and Chinese literature, thus excluding potential useful information written in other languages. Second, although to some extent there was an accord on resilience as a dynamic process (14) and leading to psychosocial outcome (49, 50), and our review also identified various resilience interventions assessed by psychosocial outcomes, there was still no consensus about the definition of resilience and proper outcome measures. Thus, there may be some resilience studies that our study did not identify, and there could be other, equally valid, ways to define resilience that we did not consider. Last but not least, this study might be limited by the selected databases. Although the investigators included the most widely used English and Chinese databases, it remains possible that some works, particularly unpublished studies conducted in other countries, were not located and examined.

## Conclusion

The study findings indicated significant benefits of resilience interventions on QOL but no significant benefits of resilience interventions on depression and neuropsychiatric behavioral symptoms among persons with neurocognitive disorders. There is an ongoing need for additional evidence to support the effectiveness of resilience interventions, how to further improve resilience interventions, how to implement them, and how to evaluate the effectiveness in persons with neurocognitive disorders. In addition, there is a need to strengthen methodological quality to assess and determine the effects of resilience interventions.

## Data Availability Statement

The original contributions presented in the study are included in the article/supplementary material, further inquiries can be directed to the corresponding author/s.

## Author Contributions

YW led the conception, data extraction, risk bias assessment, data analysis, drafting, critical review, and revision of the manuscript. YZ and WC were responsible for data search, data screening, and extraction. TL helped to proofread and edit. IC critically revised the manuscript and eventually approved the upcoming version. All authors contributed to the article and approved the submitted version.

## Conflict of Interest

The authors declare that the research was conducted in the absence of any commercial or financial relationships that could be construed as a potential conflict of interest.

## Publisher's Note

All claims expressed in this article are solely those of the authors and do not necessarily represent those of their affiliated organizations, or those of the publisher, the editors and the reviewers. Any product that may be evaluated in this article, or claim that may be made by its manufacturer, is not guaranteed or endorsed by the publisher.
